# A novel ZIKV-targeted scRNA-seq method for precise quantification of ZIKV RNA

**DOI:** 10.1128/jvi.01114-25

**Published:** 2025-09-24

**Authors:** Yang Zhou, Libo Liu, Wei Yang, Yanhua Wu, Chongyao Zhong, Yuxuan Liu, Kunqi Lin, Dongying Fan, Yisong Wang, Peigang Wang, Jing An

**Affiliations:** 1 Department of Microbiology, School of Basic Medical Sciences, Capital Medical University 352150, Beijing, China; 2 Laboratory for Clinical Medicine, Capital Medical University, Beijing, China; 3 Department of Parasitology, School of Basic Medical Sciences, Guizhou Medical University 74628, Guiyang, Guizhou, China; 4 National Center of Technology Innovation for Animal Model, State Key Laboratory of Respiratory Health and Multimorbidity, Key Laboratory of Pathogen Infection Prevention and Control (Peking Union Medical College), Ministry of Education, NHC Key Laboratory of Comparative Medicine, Chinese Academy of Medical Sciences and Peking Union Medical College 12501, Beijing, China; The Ohio State University, Columbus, Ohio, USA

**Keywords:** Zika virus, targeted single-cell RNA sequencing, 10× Genomics scRNA-seq, precise quantification, targeted cells

## Abstract

Zika virus (ZIKV), transmitted by mosquitoes, poses a serious public health threat. Currently, precise quantitative diagnostic methods are lacking. Existing single-cell RNA-sequencing (scRNA-seq) techniques are challenged to detect ZIKV RNA due to its absence of a poly(A) tail, hindering the identification of infected cells. In this study, we developed a novel ZIKV-targeted scRNA-seq method that enables precise quantification of ZIKV RNA in individual cells. Immunocompetent suckling mice were intracerebrally infected with ZIKV to establish persistent infection in the brain. Samples were collected at 10-day post infection for ZIKV-targeted and 10× Genomics scRNA-seq analysis. Comparative analysis identified 17 distinct cell types in both ZIKV-infected and control suckling mouse brains, with significant changes in cell type distribution and proportion post-infection. The ZIKV-targeted scRNA-seq method suggested higher efficiency in capturing exogenous cells compared to the 10× Genomics scRNA-seq method. Both methods identified multiple endogenous and exogenous cell types susceptible to ZIKV, with peripheral blood-derived monocytes/macrophages (PBDMMs), neurons, and T cells as primary cell types expressing ZIKV RNA. IFA validated scRNA-seq findings, revealing that neurons and microglia could be infected by ZIKV, with significant reductions in their numbers post-infection. This study presents a novel ZIKV-targeted scRNA-seq that enables accurate quantification of ZIKV RNA within individual cells, identifies key susceptible cell types, and offers advantages in detecting exogenous cells, making it a scalable solution for providing valuable insights into therapeutic and vaccine development.

**IMPORTANCE:**

This study marks the first use of a scRNA-seq method tailored for ZIKV, allowing accurate measurement of ZIKV RNA in individual cells and identification of critical susceptible cell types. A comparative analysis with the 10× Genomics scRNA-seq method highlighted the advantages of ZIKV-targeted scRNA-seq in terms of accuracy and practicality, particularly its superior ability to capture exogenous cells. Beyond ZIKV, this method also helps establish precise quantification of viral RNA at the single-cell level for other viruses by designing target-specific beads based on conserved regions of the viral genome. This advancement is set to greatly enhance studying pathogenesis of ZIKV infection and then significantly contribute to improve prevention and research in therapeutics and vaccines.

## INTRODUCTION

Zika virus (ZIKV), a *Flavivirus* transmitted primarily by *Aedes aegypti* (1, 2), has caused significant outbreaks in over 100 countries worldwide (3). ZIKV infection is often overlooked due to its typically mild clinical presentation. During the acute phase, ZIKV infection manifests with non-specific symptoms such as fever (4–6), headache (7, 8), vomiting (9), arthralgia (10–12), rash (13, 14), and lymphadenopathy (15). However, ZIKV exhibits pronounced neurotropism that leads to congenital Zika syndrome in newborns (16–19) and Guillain–Barré syndrome in adults (20–22). Previous studies showed that the viral envelope (E) protein facilitates ZIKV entry into host cells (23, 24), while the nonstructural protein 1 (NS1), NS3, and NS5 play a significant role in viral replication and immune evasion (25). Nonetheless, traditional virological approaches and bulk RNA sequencing techniques face the challenges in dissecting the dynamics of ZIKV infection, replication, and the variation in cellular susceptibility to viruses. In particular, lacking the resolution to accurately distinguish cell states across various stages of infection may hinder the comprehensive understanding of ZIKV-host interactions.

The development of single-cell RNA sequencing (scRNA-seq) in 2009 represented a major breakthrough, overcoming the limitations of traditional bulk RNA sequencing, which measures gene expression at the population level (26). By enabling in-depth and precise exploration of gene expression at the single-cell level, scRNA-seq has provided invaluable insights into biological and medical research. Over the past decade, this technology has continuously advanced our understanding of development, disease mechanisms, and cellular functions (26, 27).

However, current scRNA-seq methods, such as those developed by 10× Genomics, primarily rely on Oligo(dT)-modified magnetic beads to capture RNA from individual cells (28). Since ZIKV RNA lacks a poly(A) tail (29), it is theoretically unable to be captured by Oligo(dT)-modified beads for reverse transcription, which limits the identification and quantification of target cells during ZIKV infection in subsequent analyses, thereby restricting research on pathogenesis of ZIKV infection (30, 31).

In this study, we designed a ZIKV-targeted scRNA-seq method that enabled the precise quantification of ZIKV-infected cells and identified the majority of cell types affected during ZIKV infection. We compared the ZIKV-targeted scRNA-seq data set with data set generated by the 10× Genomics scRNA-seq method and found no significant differences in the distribution and proportion of ZIKV RNA expression between the two approaches. Additionally, we validated part of the ZIKV-targeted scRNA-seq results through immunofluorescence assay (IFA), confirming the accuracy and feasibility of this method. Our results suggested ZIKV-targeted scRNA-seq has the advantages of being cost-effective, having low environmental and equipment requirements and, thus, offers broader application prospects.

## RESULTS

### The brain of suckling mice can support ZIKV replication

**Fig 1:**
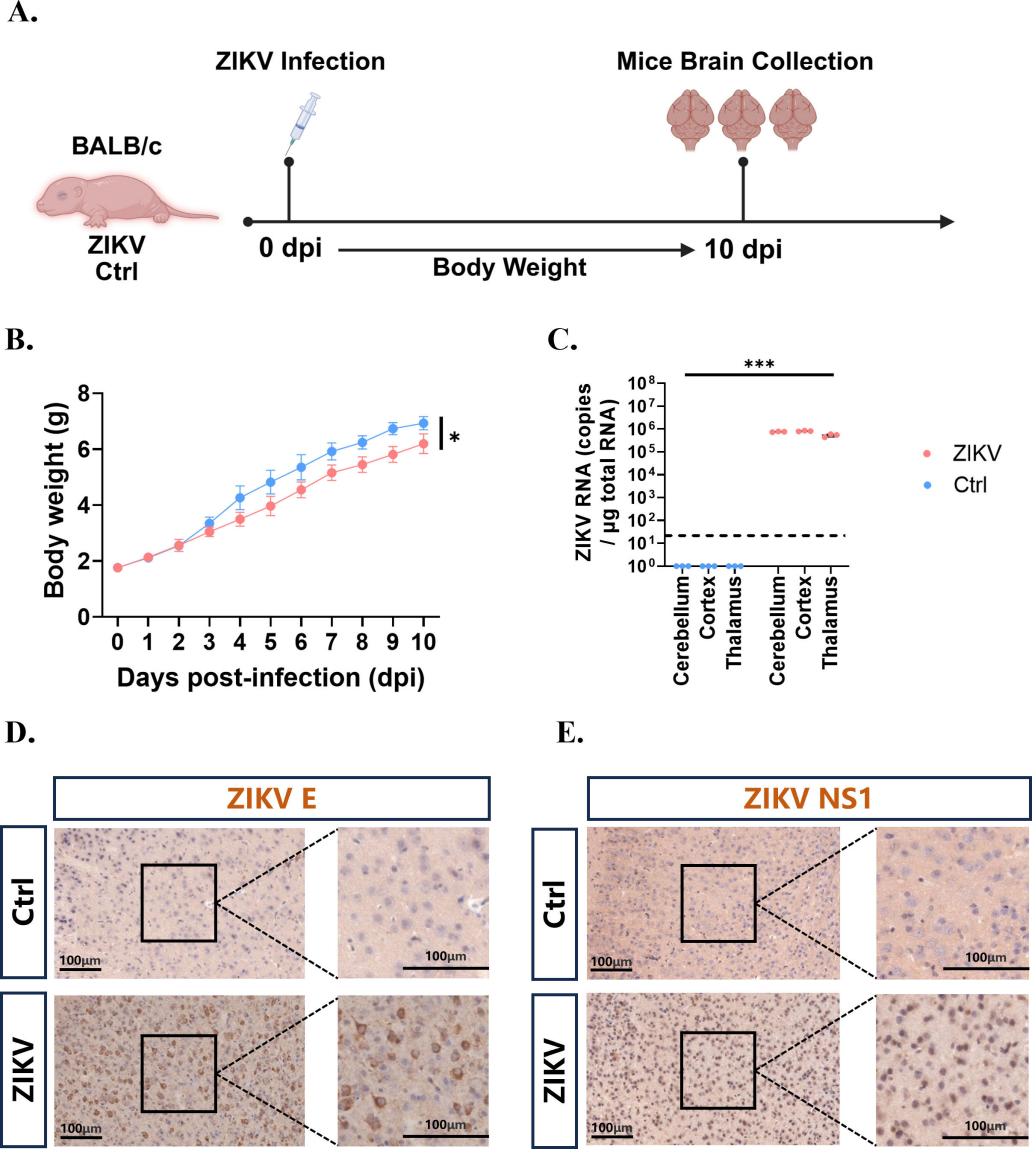
ZIKV infection in suckling BALB/c mice. (**A**) The day of ZIKV infection is designated as 0 dpi, followed by continuous monitoring of body weight of the suckling mice (*n* = 11). Mice were humanely euthanized at 10 dpi, and brains were collected. (**B**) Changes in body weight of suckling mice from 0 to 10 dpi. (**C**) Quantitative expression of ZIKV RNA in different brain regions of the suckling mice (*n* = 3). (**D**) IHC of ZIKV E antigen in the cortical region of the brains of suckling mice at 10 dpi, Scale bar, 100 µm. (**E**) IHC of ZIKV NS1 antigen in the cortical region of the brains of suckling mice at 10 dpi. Scale bar, 100 µm. Results were demonstrated as means  ±  SEM and analyzed using the two-sided Student’s *t* test. *P  <  0.05, ***P  <  0.001.

In this study, suckling mice at postnatal day 2 (P2) were intracranially infected with ZIKV to establish the immunocompetent animal model (Fig. 1A). The feasibility, similarities, and differences between ZIKV-targeted scRNA-seq and 10× Genomics scRNA-seq methods for ZIKV detection were subsequently evaluated. By 10-day post infection (dpi) following ZIKV infection, a continuous upward trend in body weight was observed in both infected- and control groups. However, a significant reduction in weight gain was noted starting from 3 dpi, with marked differences compared to the control group (*P* < 0.05) (Fig. 1B). Quantitative analysis revealed that the viral load in the brain of ZIKV-infected mice was approximately 1 × 10^6^ copies/µg by 10 dpi, with no significant differences detected among different brain regions (*P* > 0.05) (Fig. 1C). Furthermore, IHC demonstrated the presence of ZIKV E antigen (Fig. 1D) and NS1 antigen (Fig. 1E) in the brain of infected mice, thereby confirming that ZIKV can successfully infect and replicate within the brain of suckling mice.

### Design of magnetic beads and construction of a double complementary DNA library for ZIKV-targeted scRNA-seq

**Fig 2:**
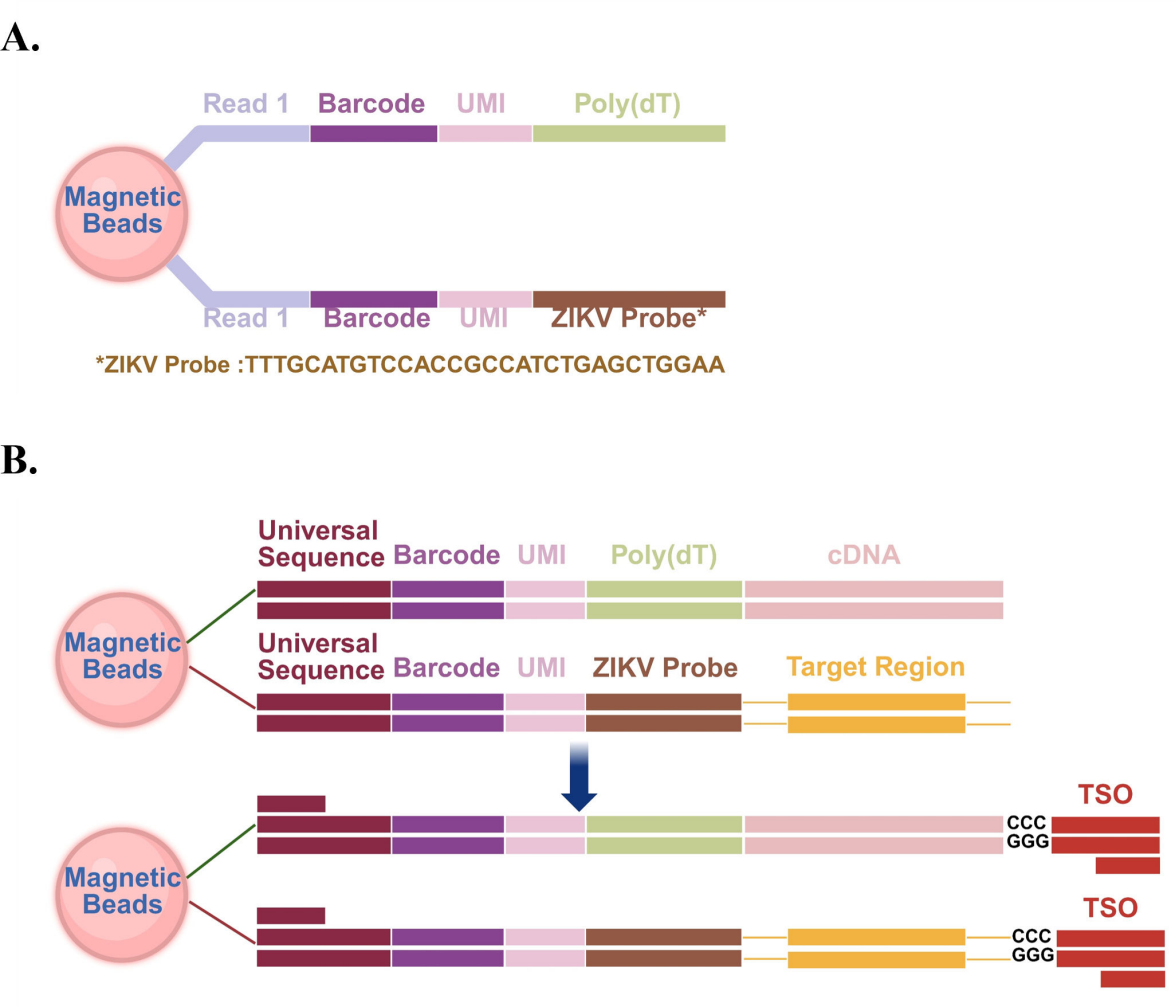
Design and strategy of ZIKV barcoding magnetic beads captured RNA. (**A**) Structure of ZIKV barcoding magnetic beads. (**B**) Diagram of reverse transcription and PCR amplification.

ZIKV-infected and control BALB/c suckling mouse brain cells were captured at 10 dpi using the FocuSCOPE microfluidic chip. control to enable targeted capture of ZIKV, we designed ZIKV Barcoding Beads incorporating Illumina Read 1 sequencing primer sequences, unique cell barcodes, unique molecular identifiers (UMIs), poly(T) nucleotide sequences, and ZIKV probes (Fig. 2A). The ZIKV probe specifically identifies ZIKV RNA, exhibiting no significant cross-reactivity with other species or virus, as confirmed by the Nucleotide Basic Local Alignment Search Tool (BLASTN). Notably, by combining the poly(T) sequence at the end of the oligonucleotide with the ZIKV-specific targeting probes, we successfully achieved precise capture and labeling of host RNA and ZIKV RNA from the brain cells of suckling mice.

To obtain a sufficient quantity of full-length complementary DNA (cDNA) products, the RNA enriched with magnetic beads underwent treatment with a template-switching oligonucleotide (TSO) during the reverse transcription process. Subsequently, the PCR amplification of the synthesized cDNA products was performed by adding PCR handle sequences (compatible with Illumina next-generation sequencing primers) to the 5′ end of the ZIKV Barcoding Beads. This approach facilitated the enrichment of full-length cDNA products (Fig. 2B).

**Fig 3:**
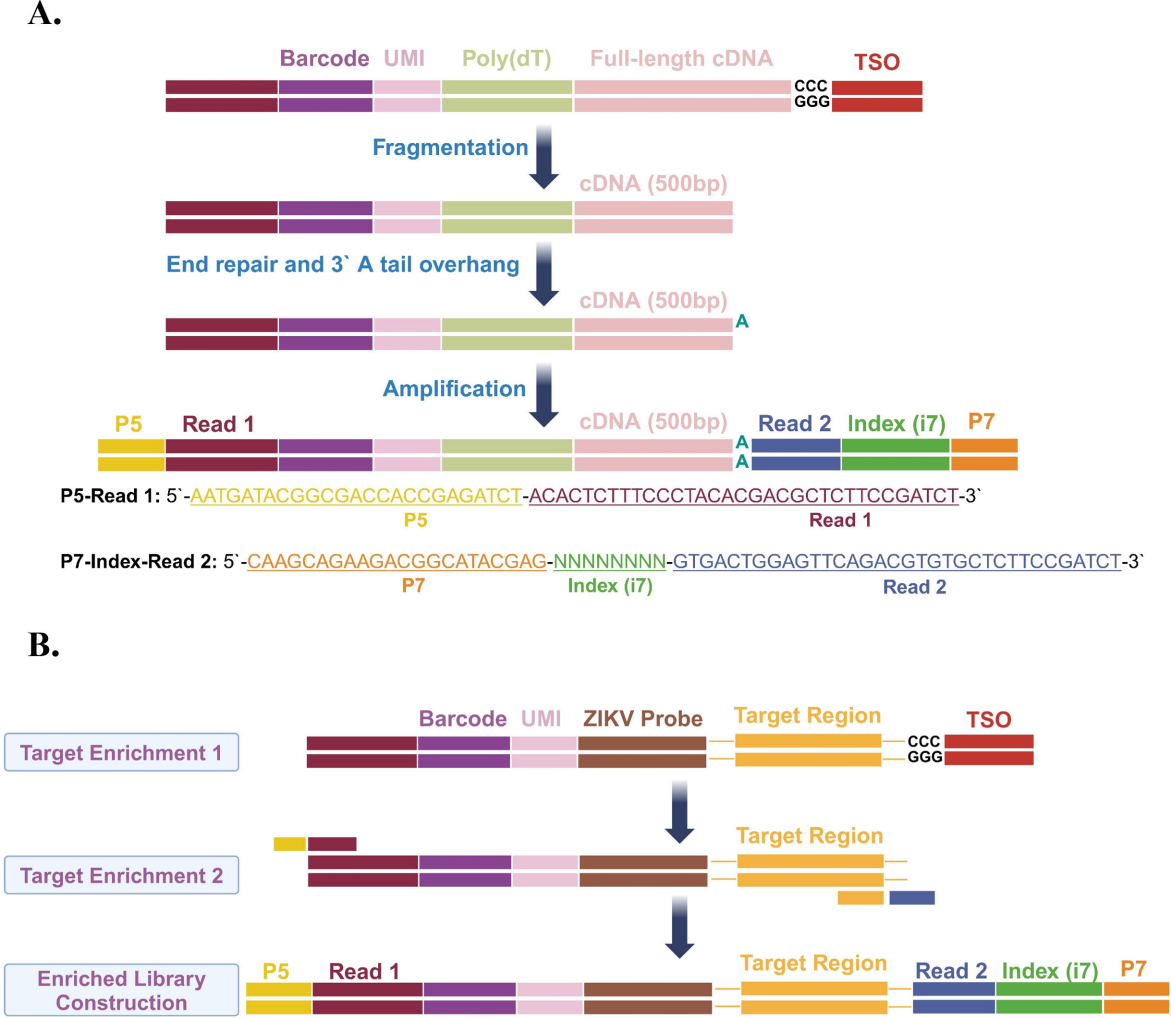
cDNA library construction of ZIKV-targeted scRNA-seq. (**A**) Process of host cDNA library construction. (**B**) Process of viral enriched library construction.

On the one hand, to meet the length requirements for the sequencing library in next-generation sequencing (NGS), the fragmentation enzymes were employed to disrupt the full-length cDNA products obtained from the reverse transcription PCR amplification. Each cDNA fragment was approximately 500 bp in length and was utilized for the construction of both the host transcriptome library and the ZIKV-targeted enrichment library. For the host transcriptome library, we first performed end repair of the fragmented cDNA and added an A-tail to the 3′ end. Next, the cDNA fragments with P5 and P7 adapters at both ends were ligated, and the index (i7) at the 5′ end of the P7 adapter was introduced through PCR amplification. Finally, by filtering out low-quality fragments and selecting high-quality fragments, a high-quality host transcriptome library was obtained (Fig. 3A).

On the other hand, to obtain precise expression for ZIKV RNA in host cells, we successfully constructed a ZIKV-targeted enrichment library using ZIKV RNA captured by ZIKV Barcoding Beads. The reverse transcription and PCR amplification on the ZIKV RNA transcripts specifically captured was performed by the probes on the ZIKV Barcoding Beads, resulting in significant enrichment of cDNA products. Subsequently, using the cDNA as a template, the enrichment of ZIKV RNA was targeted to construct the enrichment library with FocuSCOPE-specific enrichment primers successfully (Fig. 3B). By constructing high-quality host transcriptome and ZIKV libraries, the scRNA-seq was performed based on the Illumina sequencing platform.

### Two single-cell RNA sequencing methods reveal similarities and differences in cellular heterogeneity

To further investigate the similarities and differences in cell types and distribution characteristics between ZIKV-targeted and 10× Genomics scRNA-seq methods in the brain of ZIKV-infected and control suckling mice at 10 dpi, we conducted a comparative analysis of the host transcriptome data sets. First, low-quality cells and those with extreme expression levels were filtered out to obtain clean and high-quality data set. Briefly, low-quality cells were defined as those with fewer than 500 UMIs per cell, fewer than 200 detected genes, or a mitochondrial gene proportion exceeding 20%. These criteria were designed to exclude cell debris, dead cells, or doublets, thereby ensuring the reliability of downstream analyses.

The ZIKV-targeted scRNA-seq method captured 9,939 cells from the control group and 13,683 cells from the ZIKV-infected group, whereas 10× Genomics scRNA-seq captured 9,661 cells from the control group and 9,029 cells from the ZIKV-infected group, indicating a discrepancy in cell capture efficiency between the two methods.

Cell type annotation was based on the Shared Nearest Neighbor (SNN) modularity optimization algorithm (24), CellMarker 2.0 (25), and scRNA-seq/transcriptome sequencing references (26–31). Cells captured by both methods were categorized into 17 distinct clusters, showing variations in the number of cells within each cluster.

**Fig 4:**
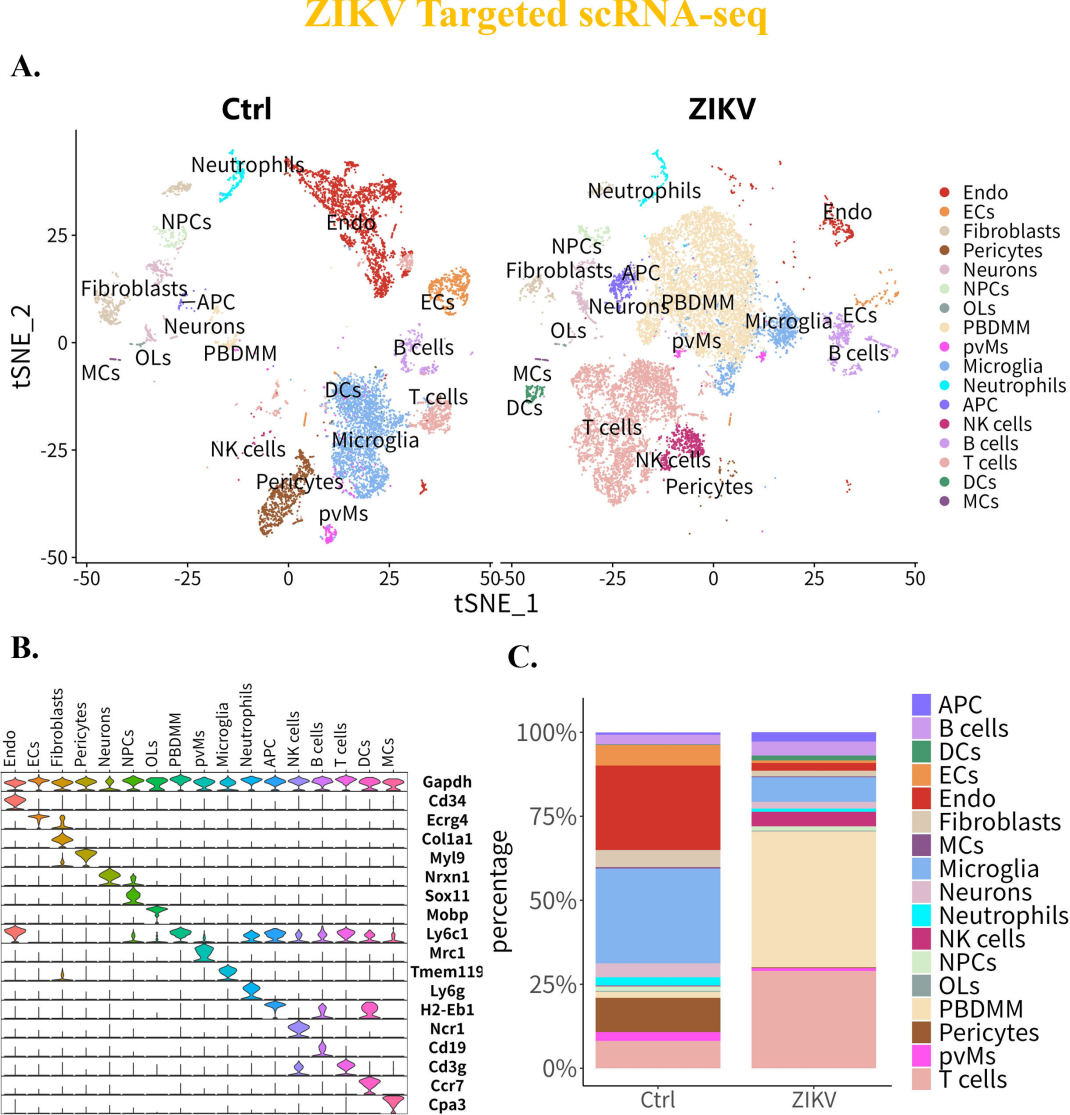
Characteristics of cell distribution in suckling mouse brain based on the ZIKV-targeted scRNA-seq. (**A**) Distribution patterns of cell types in the brain of suckling mice pre- and post-ZIKV infection. (**B**) Expression profiles of cell marker genes in different cell clusters. (**C**) Changes in cell proportions in the brain of suckling mice pre- and post-ZIKV infection.

**Fig 5:**
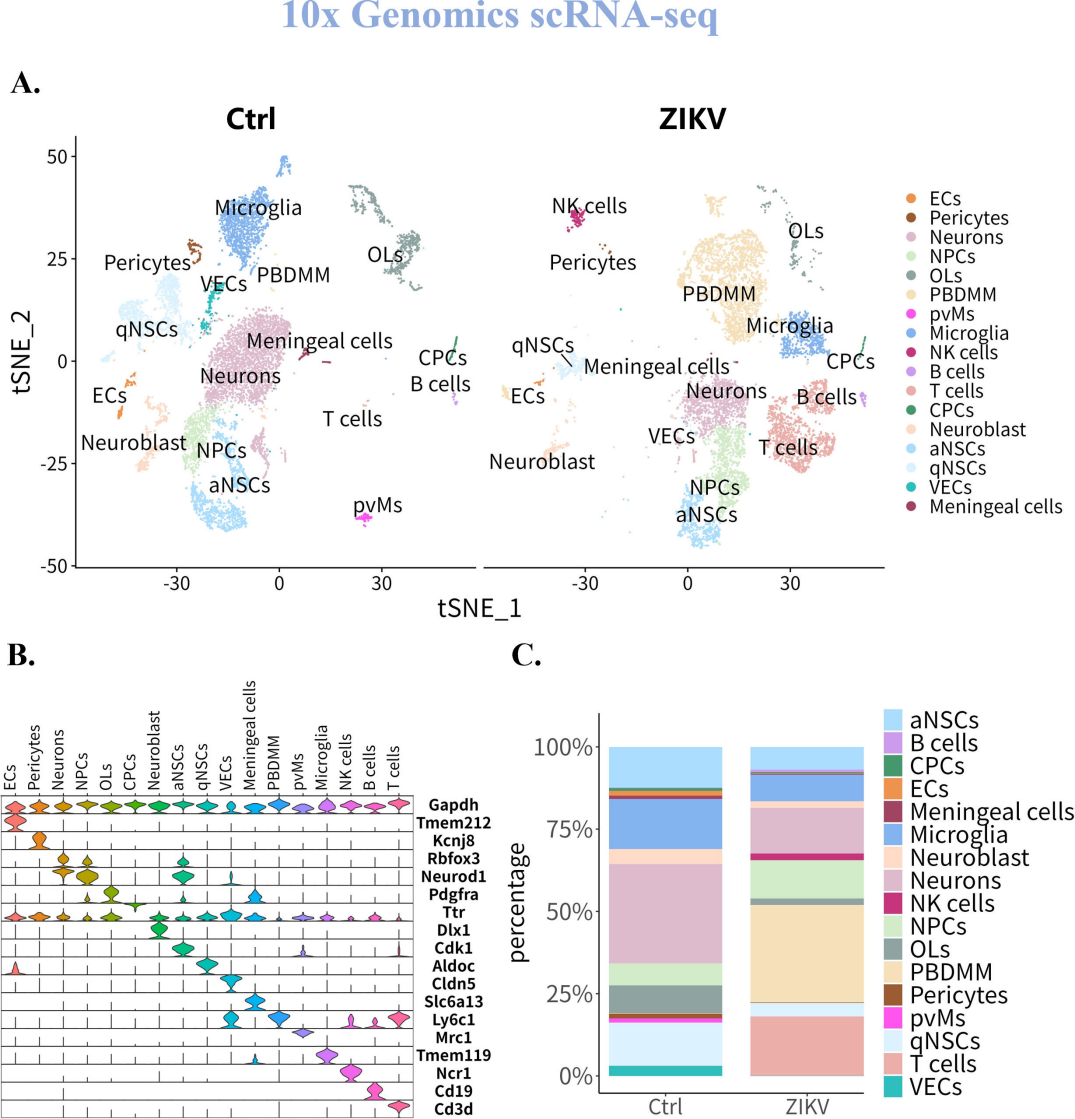
Characteristics of cell distribution in suckling mouse brain based on the 10× Genomics scRNA-seq. (**A**) Distribution patterns of cell types in the brain of suckling mice pre- and post-ZIKV infection. (**B**) Expression profiles of cell marker genes in different cell clusters. (**C**) Changes in cell proportions in the brain of suckling mice pre- and post-ZIKV infection.

In the ZIKV-targeted scRNA-seq data set, the identified cell types included Endothelial cells (Endo), Ependymal cells (ECs), Fibroblasts, pericytes, neurons, Neural Progenitor cells (NPCs), Oligodendrocytes (OLs), Peripheral Blood Derived Monocytes/Macrophages (PBDMM), Perivascular Macrophages (pvMs), microglia, neutrophils, Antigen-Presenting cells (APCs), Natural Killer (NK) cells, B cells, T cells, Dendritic Cells (DCs), and Mast cells (MCs), all of which were present both with and without ZIKV infection (Fig. 4A and B). In contrast, the 10× Genomics data set identified cell types including ECs pericytes, neurons, NPCs, neuroblasts, OLs, microglia, Activated/Quiescent Neural Stem cells (a/qNSCs), Choroid Plexus epithelial cells (CPCs), Vascular Endothelial cells (VECs), meningeal cells, PBDMMs, B cells, and T cells. Notably, NK cells were detected in the brain only after ZIKV infection, whereas pvMs disappeared following the infection (Fig. 5A and B).

Further comparative analysis (Fig. 4A and B) revealed that both methods identified a common set of cell types, including neurons, NPCs, ECs, pericytes, OLs, PBDMMs, microglia, NK cells, B cells, and T cells, suggesting overall similarity in cell-type profiles. However, the 10× Genomics data set captured a broader diversity of endogenous cells, while the ZIKV-targeted method had an advantage in detecting exogenous cells such as MCs, neutrophils, DCs, and APCs, indicating some differences in detected cell types between the two datasets.

To compare the two methods in terms of changes in cell proportions and dominant cell types, we analyzed cellular composition in both control and ZIKV-infected groups. In the ZIKV-targeted data set, Endo and microglia were the dominant cell types before infection, whereas the 10× Genomics data set indicated that neurons and microglia predominated (Fig. 4C and 5C; Table 1). Following ZIKV infection, both data sets showed a disruption of the original cell distribution, with PBDMMs becoming the predominant cell type in the brain.

**TABLE 1:** Differences in cell types and proportion detected by ZIKV targeted and 10× Genomics scRNA-seq^*a*^

Cell type	Group	Number and proportion of cells	
		ZIKV-targeted scRNA-seq	10× Genomics scRNA-seq
B cells	Ctrl	285 (2.87%)	18 (0.19%)
	ZIKV	579 (4.23%)	69 (0.76%)
ECs	Ctrl	609 (6.13%)	136 (1.42%)
	ZIKV	101 (0.74%)	21 (0.23%)
Microglia	Ctrl	2,802 (28.19%)	1,462 (15.21%)
	ZIKV	1,004 (7.34%)	739 (8.18%)
NK cells	Ctrl	25 (0.25%)	0 (0%)
	ZIKV	602 (4.40%)	186 (2.06%)
NPCs	Ctrl	129 (1.30%)	648 (6.74%)
	ZIKV	172 (1.26%)	1,058 (11.72%)
Neurons	Ctrl	416 (4.19%)	2,898 (30.16%)
	ZIKV	284 (2.08%)	1,257 (13.92%)
OLs	Ctrl	27 (0.27%)	805 (8.38%)
	ZIKV	35 (0.25%)	177 (1.96%)
PBDMM	Ctrl	182 (1.83%)	17 (0.18%)
	ZIKV	5,530 (40.42%)	2,648 (29.33%)
Pericytes	Ctrl	1,011 (10.17%)	140 (1.46%)
	ZIKV	32 (0.23%)	11 (0.12%)
pvMs	Ctrl	259 (2.61%)	121 (1.26%)
	ZIKV	112 (0.82%)	0 (0%)
T cells	Ctrl	810 (8.15%)	8 (0.08%)
	ZIKV	3,967 (28.99%)	1,632 (18.08%)
VECs	Ctrl	0	290 (3.02%)
	ZIKV	0	6 (0.07%)
aNSCs	Ctrl	0	1,186 (12.34%)
	ZIKV	0	631 (6.99%)
qNSCs	Ctrl	0	1,262 (13.13%)
	ZIKV	0	374 (4.14%)
CPCs	Ctrl	0	81 (0.84%)
	ZIKV	0	25 (0.28%)
Meningeal cells	Ctrl	0	101 (1.05%)
	ZIKV	0	16 (0.18%)
Neuroblast	Ctrl	0	436 (4.54%)
	ZIKV	0	179 (1.98%)
APCs	Ctrl	75 (0.75%)	0
	ZIKV	375 (2.74%)	0
DCs	Ctrl	18 (0.18%)	0
	ZIKV	197 (1.44%)	0
Endo	Ctrl	2,496 (25.11%)	0
	ZIKV	311 (2.27%)	0
MCs	Ctrl	42 (0.42%)	0
	ZIKV	30 (0.22%）	0
Fibroblasts	Ctrl	508 (5.11%)	0
	ZIKV	234 (1.71%)	0
Neutrophils	Ctrl	245 (2.47%)	0
	ZIKV	118 (0.86%)	0

^*a*^ Table compares cell capture between ZIKV-targeted scRNA-seq and 10× Genomics scRNA-seq. Cell types are set as cells detected by two scRNA-seq methods. Groups are identified as the Ctrl and ZIKV group.

Regarding changes in cellular composition, both scRNA-seq methods indicated a reduction in the proportions of endogenous cells, such as neurons, microglia, and NPCs post-infection, and significantly increase in exogenous cells, including T cells, B cells, NK cells, and PBDMMs. However, the 10× Genomics data set captured a higher proportion of endogenous cells (e.g., neurons, NPCs, OLs) both with or without infection, while the ZIKV-targeted method showed a greater proportion of exogenous cells, such as PBDMMs, T cells, and B cells (Fig. 4C and 5C; Table 1). This comparative analysis highlights important similarities and differences in cell capture efficiency, endogenous/exogenous cell identification between the two scRNA-seq methods, offering their respective strengths in mapping ZIKV-infected brain tissues.

### PBDMM, neuron, and T cell are the target cells in ZIKV infection

To investigate the levels of ZIKV RNA in the brain of ZIKV-infected suckling mice, a gene integration analysis was performed using ZIKV-targeted and 10× Genomics scRNA-seq data set. Data set was extracted from the ZIKV-infected group for each scRNA-seq method. In the ZIKV-targeted analysis, host data set from infected cells was merged with viral-enriched library data set, facilitating the visualization of ZIKV RNA expression across various cell types based on enriched ZIKV cDNA counts. For the 10× Genomics data set, the ZIKV cDNA sequence was directly integrated into the host data set for visualization.

**Fig 6:**
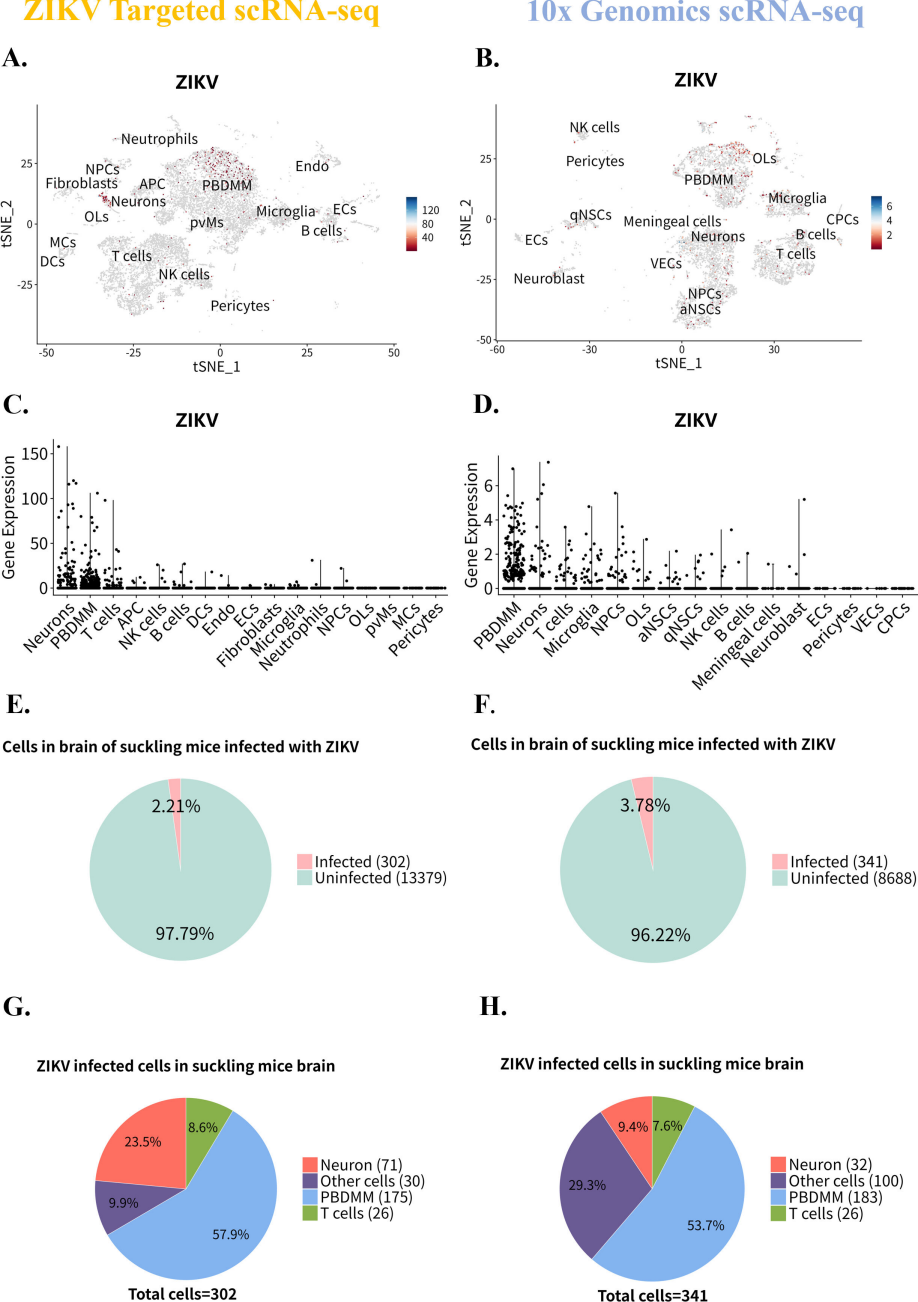
Distribution and proportion of cell expressed ZIKV RNA in suckling mouse brain infected with ZIKV. (A and B) Distribution of ZIKV RNA expression in the brain of suckling mice as captured by ZIKV-targeted scRNA-seq (**A**) and 10× Genomics scRNA-seq (**B**). (C and D) Expression levels of ZIKV RNA in different cell types identified in the ZIKV-targeted scRNA-seq data set (**C**) and 10× Genomics scRNA-seq data set (**D**). (E and F) Proportion of ZIKV RNA-expressing cells in the brain of suckling mice captured by ZIKV-targeted scRNA-seq (**E**) and 10× Genomics scRNA-seq (**F**). (G and H) Numbers and proportions of primary cell types expressing ZIKV RNA in the ZIKV-targeted scRNA-seq data set (**G**) and 10× Genomics scRNA-seq data set (**H**).

Our analysis for ZIKV targeted scRNA-seq data set revealed predominant ZIKV RNA expression in endogenous cell types, including neurons, Endo, and microglia, as well as exogenous cells, such as PBDMM, T cells, and DCs. Notably, OLs, pvMs, MCs, and pericytes showed no detectable ZIKV RNA expression (Fig. 6A). In the 10× Genomics data set, ZIKV RNA was similarly expressed in a range of endogenous cells, including neurons and NPCs, as well as exogenous cell PBDMM. However, in the 10× Genomics data set, ECs and CPCs exhibited no expression (Fig. 6B), which is different from ZIKV targeted scRNA-seq data set. Despite some differences in cell type identification between the two dataset, consistent ZIKV RNA expression was observed in neurons, PBDMM, T cells, NK cells, B cells, microglia, and NPCs, with no expression in pericytes in either data set.

Further analysis identified PBDMM, neurons, and T cells as primary ZIKV infection targets. The ZIKV-targeted data set indicated that neurons accounted for the highest proportion of ZIKV RNA expression (Fig. 6C), whereas the 10× Genomics data set showed that PBDMM was identified the highest expression levels (Fig. 6D). Furthermore, the proportions of cells expressing ZIKV RNA were investigated to gain deeper insights into ZIKV RNA expression in specific cell types (Table 2). In the ZIKV-targeted data set, 13,681 cells were analyzed, with approximately 2.21% (*n* = 302) expressing ZIKV RNA (Fig. 6E). In the 10× Genomics data set, out of 9,029 analyzed cells, approximately 3.78% (*n* = 341) tested positive for ZIKV RNA (Fig. 6F). In terms of the absolute number of cells expressing ZIKV RNA, PBDMMs were indicated by both scRNA-seq methods, followed by neurons, T cells, and other cell types (Fig. 6G and H).

**TABLE 2:** Differences in primary cell expression ZIKV RNA between ZIKV targeted and 10× Genomics scRNA-seq

Cell type	Number and proportion of cells expressing ZIKV RNA	
	ZIKV-Targeted scRNA-seq	10× Genomics scRNA-seq
PBDMM	175 (3.18%)	183 (6.95%)
Neurons	71 (25%)	32 (2.5%)
T cells	26 (0.66%)	26 (1.59%)
Microglia	5 (0.50%)	31 (4.2%)
NPCs	3 (1.74%)	26 (2.46%)
OLs	0	11 (6.21%)
B cells	8 (1.38%)	2 (2.90%)
NK cells	4 (0.66%)	6 (3.23%)

**Fig 7:**
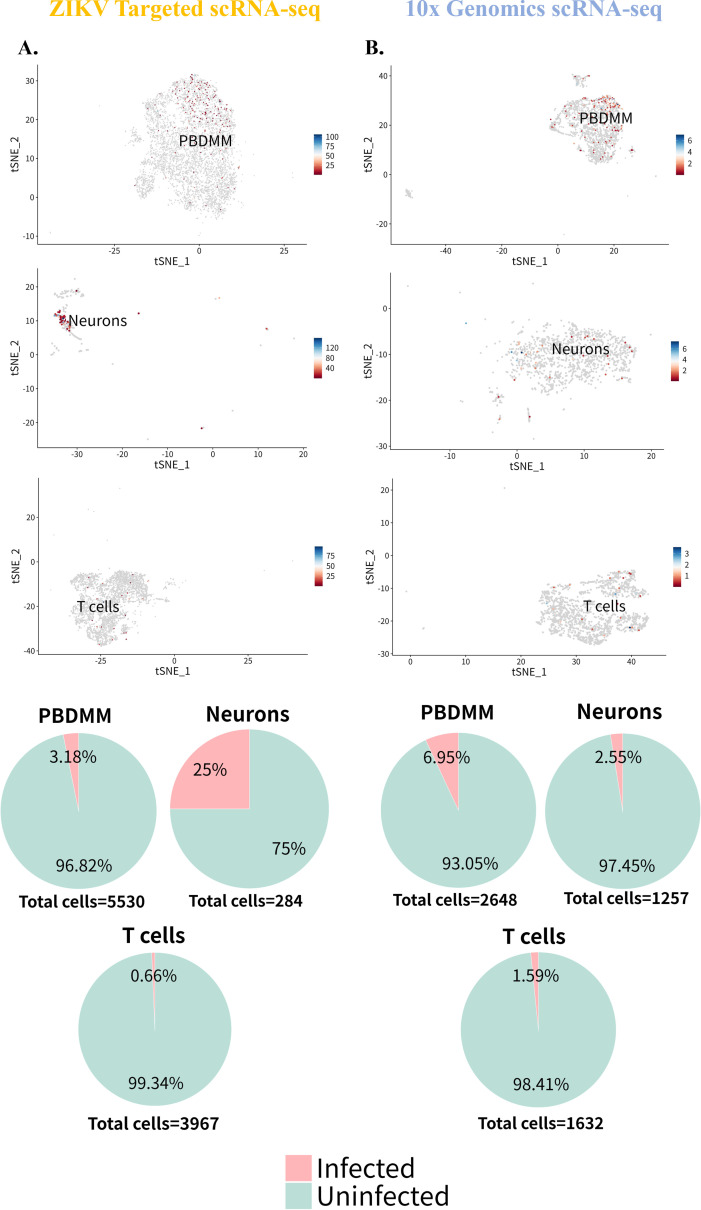
PBDMM, neurons, and T cells expressing ZIKV RNA in suckling mouse brain infected with ZIKV. (**A**) Distribution and proportions of PBDMMs, neurons, and T cells expressing ZIKV RNA in the ZIKV-targeted scRNA-seq data set. (**B**) Distribution and proportions of PBDMMs, neurons, and T cells expressing ZIKV RNA in the 10× Genomics scRNA-seq data set.

Subsequently, the PBDMM, neurons, and T cells were analyzed independently to elucidate their expression characteristics regarding ZIKV RNA. The proportion of PBDMM expressing ZIKV RNA was not significantly different across data set. In the ZIKV-targeted data set, 3.18% (176 cells) of 5,530 PBDMMs expressed ZIKV RNA, predominantly located in the central and upper regions of the cell cluster (Fig. 7A; Table 2). In the 10× Genomics data set, 6.95% (183 cells) of 2,648 PBDMMs expressed ZIKV RNA, concentrated in the upper right quadrant of the cell cluster (Fig. 7B; Table 2). For neurons, the ZIKV-targeted scRNA-seq data set indicated that 25% (71 cells) of 284 neurons expressed ZIKV RNA, primarily on the right side of the cell cluster (Fig. 7A; Table 2), whereas the 10× Genomics data set showed that 2.55% (32 cells) of 1,257 neurons expressed ZIKV RNA, displaying a scattered distribution (Fig. 7B; Table 2). Regarding T cells, 0.66% (26 cells) of 3,967T cells expressed ZIKV RNA in the ZIKV-targeted data set (Fig. 7A; Table 2), compared to 1.59% (26 cells) of 1,632T cells in the 10× Genomics scRNA-seq data set (Fig. 7B; Table 2). However, it is worth noting that the absolute number and proportion of cells carrying ZIKV RNA remained unchanged.

### Neuron and microglia could be infected by ZIKV

**Fig 8:**
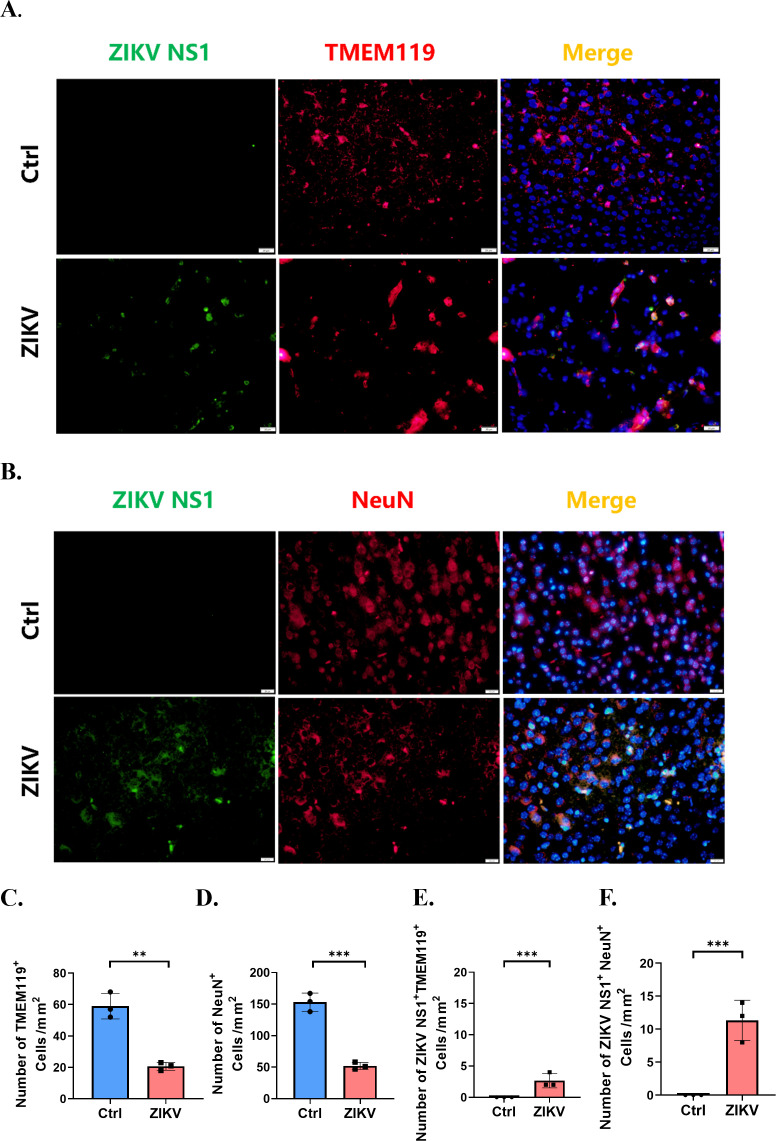
IFA for ZIKV NS1, TMEM119, and NeuN antigens. (**A**) IFA for ZIKV NS1 and TMEM119 antigens, quantification of TMEM119 positive cells, and co-localization of ZIKV NS1 with TMEM119 positive cells, Scale bar, 20 µm. (**B**) IFA for ZIKV NS1 and NeuN antigens, quantification of NeuN-positive cells, and co-localization of ZIKV NS1 with NeuN-positive cells, Scale bar, 20 µm. (**C and D**) The number of TMEM119(C)/NeuN(D) positive cells/mm^2^ between ZIKV infection and control groups. (**E and F**) The number of co-localization of ZIKV NS1 and TMEM119(E)/NeuN(F) positive cells/mm^2^ between ZIKV infection and ctrl groups. Results were demonstrated as means  ± SEM and analyzed using the two-sided Student’s *t* test. **P  <  0.01, ***P  <  0.001. Nuclei were stained with DAPI.

To validate the scRNA-seq findings on the susceptibility of neurons and microglia to ZIKV infection, IFA tests were conducted for ZIKV NS1 antigen, along with the neuronal marker NeuN and microglial marker TMEM119, respectively (Fig. 8A and B). Following ZIKV infection, we observed a significant reduction in staining intensity for TMEM119 (Fig. 8C) and NeuN (Fig. 8D) compared to the control groups, with minimal co-localization between ZIKV NS1 and these cellular markers (Fig. 8E and F). These results corroborate our findings from scRNA-seq data set, indicating that ZIKV infection damages neurons and microglia and reduces the cellular number in the suckling mouse brain, further confirming that both cell types are susceptible to ZIKV infection.

### ZIKV-targeted scRNA-seq method has multiple advantages

The value and scenarios of the ZIKV-targeted scRNA-seq method were assessed from multiple dimensions (Table 3). First, the ZIKV-targeted scRNA-seq supports manual operation for single-cell sorting, and the FocuSCOPE microfluidic chip is portable due to the lightweight and small-sized. In contrast, while the 10× Genomics scRNA-seq was allowed to complete single-cell sorting automatically via the 10× Genomics Chromium Single-Cell 3′ kit, the equipment is heavy and bulky and requires high stability, which makes frequent transportation inconvenient and restricts it to fixed locations.

**TABLE 3:** Application and features between ZIKV-targeted and 10× Genomics scRNA-seq

Index	ZIKV-targeted scRNA-seq	10× Genomics scRNA-seq
Single-cell sorting methods	FocuSCOPE microfluidic chip (manual processing)	10× Genomics Chromium Single-Cell 3′ kit (automatic processing)
Key features of single-cell sorting	Lightweight, small-sized, portable	Heavy, large-sized
Main equipment in sample preparation before library construction	Automated cell counter, centrifuge (15/50 mL), thermal cycler, metal bath	Automated cell counter, centrifuge (15/50 mL), 10× Genomics Chromium
Features of equipment in sample preparation before library construction	Low cost (approximately $28,000), portable	High cost (approximately $41,000)
Sequencing platform	NovaSeq 6000	Novaseq 6000
Application scenarios	Versatile and timeliness (laboratory, remote regions, or field investigation)	Restricted (laboratory)

Second, both methods require a horizontal centrifuge suitable for 15–50 mL centrifuge tubes and an automated cell counter to obtain high-quality single-cell suspensions before library construction. In addition, the 10× Genomics scRNA-seq completes these steps in an integrated workflow via the Chromium Single-Cell 3′ kit including mRNA reverse transcription and PCR amplification, whereas the ZIKV-targeted scRNA-seq relies on a metal bath and thermal cycler. However, in terms of equipment costs, the ZIKV-targeted scRNA-seq is significantly less expensive than the 10× Genomics method. Moreover, both methods can ensure high-quality sequencing data based on the Illumina sequencing platform, while the thermal cyclers and metal baths are becoming increasingly miniaturized and portable currently, which indicates the ZIKV-targeted scRNA-seq combining low cost and device portability, is suitable for laboratories, field investigations, and single-cell transcriptomics in remote areas, demonstrating considerable practical application value. In contrast, the 10× Genomics scRNA-seq method still depends on a fixed laboratory environment, limiting its application scenarios. Although tissue cryopreservation solutions have been developed and applied to transport fresh tissues, they may significantly affect intracellular gene expression profiles and cell viability due to mechanical forces.

## DISCUSSION

ZIKV is primarily transmitted through mosquito bites and continues to pose a major public health threat globally. Since 2015, large-scale outbreaks in Brazil, Colombia (32), and Puerto Rico have been associated with a surge in congenital Zika syndrome (CZS) among neonates and Guillain-Barré syndrome (GBS) in adults (33, 34), raising serious concerns about its long-term neurological impact. Mechanistic studies suggest that ZIKV hijacks peripheral blood monocytes to traverse the blood-brain barrier, subsequently infecting both central and peripheral nervous systems (35). This neurotropism results in the infection and apoptosis of neural cells, ultimately leading to severe and often irreversible neurological damage (36–38). Moreover, recent studies have revealed potential associations between ZIKV infection and various host cellular events, including cell cycle arrest (39), DNA damage (40), mitochondrial fragmentation (41), and endoplasmic reticulum (ER) stress (42). However, the pathogenesis of ZIKV infection is highly complex. The intricate regulatory networks and cellular heterogeneity within host tissues become the formidable challenges to traditional virological methods and bulk RNA sequencing.

In recent years, the emergence of scRNA-seq technology has transformed virological research, offering unparalleled insights into host-virus interactions and identifying specific cell types infected by viruses (43–45). However, ZIKV, as the member of RNA viruses, was used to be considered poorly captured by oligo(dT)-based methods due to their lack of poly(A) tails (46). Interestingly, our previous study demonstrated that oligo(dT)-modified beads were capable of detecting and quantifying ZIKV RNA in the mouse testis by integrating the ZIKV genome sequence into the host transcriptome data set during downstream analysis (47). However, the specificity in oligo(dT)-based capture of ZIKV RNA and the underlying mechanisms remain to be clarified, highlighting the urgent need for the development of a ZIKV-specific scRNA-seq method to overcome the current limitations in ZIKV research.

In this study, we developed a novel ZIKV-targeted scRNA-seq method that accurately quantifies ZIKV RNA within individual cells, revealing key cell types susceptible to infection and offering a refined understanding of ZIKV infection dynamics. The comparative analysis between the ZIKV-targeted scRNA-seq and the 10× Genomics scRNA-seq methods highlights significant differences in cell capture efficiency. Specifically, the ZIKV-targeted method exhibited higher efficacy in capturing exogenous cells, such as PBDMM, T cells, and MCs, whereas the 10× Genomics scRNA-seq method demonstrated broader coverage of larger endogenous cells, including neurons and glial cells (Table 1). The characteristics of cell type indicate that there is a difference in cell capture efficiency between the two methods. Depending on the research objective, different scRNA-seq methods can be chosen.

Possible reasons underlying the differences in cell types captured by the two methods include several technical factors. First, a 70 µm filter was used to process the dissociated single-cell suspension from mouse brains in the ZIKV-targeted scRNA-seq method, manually, which can effectively block larger cells and debris (Table 3). In ZIKV-infected suckling mouse brains, the majority of small-sized cells are infiltrating exogenous cells. Therefore, this method yields a higher capture rate of these exogenous cells compared to the automated dissociation and cell capture process performed by the 10× Genomics scRNA-seq method. This represents one of the primary factors contributing to the observed differences in cell type composition between the two methods.

Additionally, in terms of sample preparation, the ZIKV-targeted scRNA-seq method involves manually injecting the single-cell suspension into a microfluidic chip, where individual cells are randomly distributed into wells based on a Poisson distribution (Table 3). ZIKV barcoding beads and lysis buffer are then manually introduced to capture both cells and their mRNA. In contrast, the 10× Genomics scRNA-seq method relies on the automated 10× Chromium system for cell encapsulation and capture (Table 3), indicating that the differences in sample preparation workflows may contribute to variations in both cell number and cell type representation. Notably, the manual approach allows for greater control over sample handling and offers a gentler, customizable alternative to automated systems. Regarding capture efficiency, the ZIKV barcoding beads used in the targeted method may possess higher RNA capture efficiency compared to those used in the 10× Genomics scRNA-seq method. This may further contribute to the discrepancies in cell capture rates and cellular diversity observed between the two methods.

Given that the ZIKV-targeted scRNA-seq consistently captured a greater number of cells in both infected and control groups, we conclude that the differences in cell number and composition are primarily driven by technical variation between the methods rather than underlying biological differences.

Our findings showed that ZIKV RNA was detected in various cell types, primarily expressed in neurons, PBDMM, and T cells using both methods, while the capture mechanisms of ZIKV RNA in 10× Genomics scRNA-seq were not clear. It is speculated that this may be associated with the potential post-infection modifications of viral RNA, such as polyadenylation, which warrant further investigation. These modifications may not be mere artifacts of viral gene expression but could play crucial roles in host-virus interactions, which need further investigation. Considering the specificity of the ZIKV probe, ZIKV-targeted scRNA-seq method demonstrates scientific validity and representativeness in quantifying intracellular ZIKV RNA expression and identifying target cell types.

Despite these advantages, a limitation of the ZIKV-targeted scRNA-seq method is the exclusion of larger cells, such as neurons and glial cells, during the filtration process. This could lead to a biased representation of certain cell populations. Future studies may benefit from combining this approach with other technologies, such as flow cytometry or traditional Oligo(dT)-based capture methods, to achieve a more comprehensive representation of both small and large cell types. Such integration would further enhance the versatility and applicability of the ZIKV-targeted method.

Although cell preservation solutions have been developed to maintain cell viability during long-distance transport; however, external factors, such as mechanical vibrations and temperature fluctuations, may compromise genomic stability and cellular phenotypes (48, 49). It can be hypothesized that long-distance transport may increase the risk of phenotypic and genomic changes in cells. Such changes could undermine the accuracy of experimental outcomes and negatively affect the correct identification of cell types and their corresponding ZIKV RNA expression levels in downstream analyses. By employing a manual approach for cell sorting, cDNA reverse transcription, PCR amplification, and library construction, the ZIKV-targeted scRNA-seq method circumvents these challenges and allows to perform on-site, which significantly reduces the risks of physical and genomic changes during transport and offers a more affordable and accessible alternative (Table 3). This characteristic is particularly crucial in regions where the cost and availability of advanced laboratory infrastructure pose significant barriers to effective viral surveillance and provide a feasible solution in diverse research scenarios.

In addition to identifying ZIKV-infected cell types, the ZIKV-targeted scRNA-seq method is able to quantify viral RNA expression across diverse cell populations provides critical insights into how ZIKV interacts with different cellular environments. These findings can shed light on potential mechanisms of immune evasion or persistence within the host, which are key factors in developing targeted therapeutic interventions. For instance, understanding the viral load in neurons and glial cells could offer valuable data for the development of neuroprotective therapies in ZIKV-infected patients. Additionally, the data generated by the ZIKV-targeted scRNA-seq method could play a pivotal role in future vaccine development, particularly in designing vaccines that target specific cell types or tissues most affected by ZIKV infection. Further research could explore how this information could be leveraged to develop cell-specific immune responses, offering broader applicability not only for ZIKV but also for related viruses.

Overall, the ZIKV-targeted scRNA-seq method offers a broadly applicable and cost-effective strategy for studying host-ZIKV interactions, with implications for both therapeutic and vaccine development. Future work can build upon these findings to further explore viral mechanisms and improve global responses to ZIKV and related viruses. However, given the absence of reported ZIKV infection cases in China since 2019, a limitation of this method is the challenge of validating it with clinical samples from ZIKV-infected individuals. Nonetheless, with the anticipated advancement of international collaborations in vector-borne infectious disease prevention and control, this method holds promise for eventual clinical validation in ZIKV-endemic regions.

## MATERIALS AND METHODS

### ZIKV infection animal model

BALB/c suckling mice at postnatal day 2 were infected with the SMGC-1 ZIKV strain via intracranial injection with an infection dose of 20 µL containing 100 PFU. The mice were humanely euthanized at the 10 dpi, and brain tissues were collected for further analysis.

### Preparation of single-cell suspensions from suckling mouse brain

Suckling mouse brain tissues were minced into 1 mm^3^ pieces using ophthalmic scissors and digested in Collagenase IV (SIGMA, USA) under the conditions of 37°C, 250 rpm shaking for 15 min. The digested brain tissue was filtered through a 70 µm mesh to collect the cell suspension. The cell suspension was then resuspended in calcium- and magnesium-free 1× PBS and washed by centrifugation at 350 g, 4°C, for 5 min with no brake. According to the manufacturer’s instructions, the SCelLive Debris Removal Kit (Singleron Biotechnologies, China, 13200066) was used to remove cell debris and dead cells from the cell suspension. Cell viability was assessed using AOPI staining (Counter Star, China,RE010212), and samples with cell viability ≥85% and cell aggregation rate ≤10% were deemed suitable for further single-cell library preparation.

### 10× Genomics scRNA-seq

According to the manufacturer’s instructions for the 10× Genomics Chromium Single-Cell 3′ Kit (V3), the single-cell suspensions were loaded onto the 10× Chromium system to capture individual cells. Subsequent cDNA amplification and library construction steps were performed according to the standard protocol. The libraries were sequenced on the Illumina NextSeq 500 platform (paired-end, 150 bp) at LC-Bio Technology Co., Ltd. (Hangzhou, China), using a multiplexed sequencing run.

### ZIKV-targeted scRNA-seq

We collaboratively developed a Zika virus (ZIKV)-targeted single-cell RNA sequencing kit with Singlera Genomics Co., Ltd. and performed sorting and cDNA library construction on single cells derived from the brains of suckling mice. To overcome the gap in current ZIKV-host interactions analysis methods in sc-RNA seq, probes binding to ZIKV sequence and oligo-dT were added to the magnetic capture beads, which allows to capture and reverse transcribing mRNA from host and virus sequence. The ZIKV probe was designed to map current ZIKV lineages based on the National Center for Biotechnology Information (NCBI). The Nucleotide Basic Local Alignment Search Tool (BLASTN) was applied to evaluate the specificity of ZIKV Probe. Following the manufacturer’s recommendations, the single-cell suspension was briefly added to a microfluidic chip, introducing ZIKV-targeted modified capture magnetic beads (ZIKV Probe: TTTGCATGTCCACCGCCATCTGAGCTGGAA) along with cell lysis buffer into the microfluidic chip to capture single cells and RNA. The mixture was then incubated in a heated metal bath for cDNA reverse transcription. Subsequently, cDNA products were amplified using the 7500 Real-Time PCR System (Applied Biosystems, USA). After purification with Ampure XP magnetic beads (Beckman Coulter Life Sciences, A63880, USA), the purified cDNA amplification products were used for constructing GEXSCOPE single-cell transcriptomic libraries for both host and virus. Quality control of the cDNA libraries was performed using a Qubit 4.0, and sequencing was conducted based on the Illumina PE150 strategy on the Novaseq platform.

### The quality control of raw data set for 10× Genomics scRNA-seq

Sequencing results were demultiplexed and converted to FASTQ format using the Illumina bcl2fastq software. Sample demultiplexing, barcode processing, and single-cell 3′ gene counting were performed using Cell Ranger v5.0.1, and the scRNA-seq data set was aligned to the Ensembl genome GRCm38 reference genome (release-95). The Cell Ranger output was subsequently loaded into Seurat v4.1.1 for dimensionality reduction, clustering, and analysis of the scRNA-seq data set. In total, 22,988 cells passed the quality control thresholds: genes expressed in fewer than one cell were removed, a low cutoff of more than 500 genes expressed per cell was applied, and the percentage of expression derived from mitochondrial DNA was kept below 25%.

### The primary analysis of data set for ZIKV-targeted scRNA-seq

Raw reads were processed to generate gene expression profiles using CeleScope v2.0.6 (Singleron Biotechnologies) with default parameters. After pro-processing, R2 reads were aligned against the hg38 transcriptome using STAR v2.6.1a. Uniquely mapped reads were then assigned to exons with FeatureCounts (v2.0.1). Successfully Assigned Reads with the same cell barcode, UMI and gene were grouped together to generate the gene expression matrix for further analysis. ZIKV-enriched libraries were analyzed using CeleScope v2.0.6 (Singleron Biotechnologies). After pro-processing, R2 reads were aligned against the ZIKV genome using STAR v2.6.1a with FilterMatchNmin set to 80. After obtaining the BAM file, in order to remove ambient contamination, we adopted a two-step filtration process. First, a UMI needs to be supported by a certain number of reads. Second, an ZIKV-positive cell needs to be supported by a certain number of UMIs. Thresholds for supporting reads and UMIs were determined by the Otsu’s method.

### Downstream data set analysis

For the host and virus data set from ZIKV-targeted scRNA-seq, we utilized the Seurat package for data set merging, dimensionality reduction, clustering, and analysis. During quality control, we removed all genes expressed in fewer than one cell, set the minimum number of genes expressed per cell to 500, and excluded cells with more than 25% of gene expression derived from mitochondrial DNA. A total of 23,622 cells passed the quality control thresholds. To visualize the data set, we applied the LogNormalize method from the Seurat package to normalize the expression values for all 23,622 cells. The normalized expression values were then subjected to PCA (principal component analysis), utilizing the top 50 principal components for clustering and t-SNE analysis. For determining clusters, we employed the weighted SNN graph-based clustering method. Marker genes for each cluster were identified using the FindAllMarkers function in the Seurat package, based on the Bimod maximum likelihood ratio test, with identification criteria set to genes expressed in more than 10% of the cells in a cluster and an average log (Fold Change) greater than 0.25. Bar plots and violin plots were generated using the ggplot2 R package v3.4.0.

### RT-qPCR

The RNA of suckling mouse brains was initially extracted according to the manufacturer suggestions (Transgen China, ET101-01). Then, the RNA was performed by one-step RT-qPCR (Tiangen, China) on 7500 Real-Time PCR System (Applied Biosystems, USA) to detect the ZIKV RNA in each brain region of suckling mice. The primer sequences were as follows: ZIKV forward: 5′-TCAGACTGCGACAGTTCGAGT-3′; ZIKV reverse: 5′-GCATATTGACAATCCGGAAT-3′. The ZIKV RNA loading was quantitated by GraphPad prism software (Version 9.0.0).

### IFA

The suckling mouse brains were fixed overnight in a modified Davidson’s fixative solution (30 mL of 40% formaldehyde, 15 mL of ethanol, 5 mL of glacial acetic acid, and 50 mL of distilled water), while other tissues were fixed overnight in a 4% paraformaldehyde (PFA) solution before being dehydrated and embedded in paraffin. Sections (5 µm thick) were incubated overnight at 4°C with the following primary antibodies: mouse anti-ZIKV antibody NS1 (Invitrogen, California, USA, 1:500), rabbit anti-Tmem119 antibody (Cell Signaling Technology, Danvers, USA, 1:400), and rabbit anti-NeuN antibody (Abcam, Oxford, UK, 1:400). After washing with PBS, the sections were incubated for 1 h at 37°C with the following secondary antibodies: donkey anti-mouse IgG (Alexa Fluor 488, Life Technologies, 1:1,000) and donkey anti-rabbit IgG (Alexa Fluor 594, Life Technologies, 1:1,000). Images were captured using an Olympus IX71 microscope (Olympus, Japan).

### Immunohistochemistry

To investigate the distribution of ZIKV E^+^ and ZIKV NS1^+^ cells in the cortical regions of suckling mouse brains, brain sections were incubated overnight at 4°C with mouse anti-ZIKV NS1 monoclonal antibody (Invitrogen, California, USA, 1:500) and mouse anti-ZIKV 4G2 (1:500) as primary antibodies. After washing with PBS, the sections were stained with a secondary antibody, HRP-conjugated goat anti-mouse antibody (Zhongshan Golden Bridge Biotechnology Co., Ltd., China) for 1 h. The reaction was visualized by adding 3,3′-diaminobenzidine (DAB) as the chromogenic substrate, and the reaction was terminated by removing DAB.

### Cell quantification

The positive staining cells in the IFA were analyzed using ImageJ v1.8.0.112 software, with each group comprising at least three suckling mice. For each mouse, sections were analyzed across a minimum of five fields of view (×400 magnification), with each field containing 100 or more cells. The cell count was expressed as the number of cells per mm².

### Data set statistics and reproducibility

Images from the IFA were obtained from at least three independent experiments. All statistical analyses were conducted using SPSS version 17.0 software (IBM, Armonk, NY, USA) and were subsequently verified using Microsoft Excel 2016. A two-tailed Student’s *t*-test was employed to analyze quantitative data set between two groups that followed a normal distribution. A *P*-value of less than 0.05 was considered statistically significant between the two groups.

## Data Availability

The ZIKV-targeted and 10× Genomics scRNA-seq data are available in the NCBI database under BioProject accession number PRJNA1306354 and Sequence Read Archive accession numbers SRR34999096–SRR34999101, SRR35181684.
